# Do I really need to be admitted? A case for more outpatient therapies for acute decompensated heart failure

**DOI:** 10.1007/s10741-025-10581-4

**Published:** 2025-12-02

**Authors:** Alexander G. Hajduczok, Andrew J. Sauer, Nicholas Wettersten

**Affiliations:** 1 Division of Cardiology, University of California San Diego Health, CA La Jolla, USA; 2 Saint Luke’s Mid America Heart Institute, University of Missouri-Kansas City, Kansas City, MO USA; 3 Division of Cardiovascular Medicine, San Diego Veterans Affairs Medical Center, CA San Diego, USA

Acute decompensated heart failure (ADHF) drives more than 1.2 million hospitalizations annually, accounting for approximately 40% of the estimated $32 billion annual U.S. expenditure on heart failure (HF) care [1]. Beyond hospital admissions, emergency and urgent outpatient visits account for nearly half of worsening HF events [2]. Despite advances in medical optimization with guideline-directed medical therapy (GDMT), the management of congestion remains the leading driver of resource utilization and rehospitalization.

Historically, hospitalization for ADHF has been the default setting for decongestion because the scalability and effectiveness of outpatient options remain limited. Intravenous (IV) loop diuretic therapy is logistically difficult outside the hospital, as close monitoring for safety and efficacy, as well as significant time investment, are required. As a result, many patients who could be safely diuresed in the outpatient setting are frequently admitted out of convenience and lack of viable alternatives. There are substantial gaps in our ability to achieve and maintain decongestion, especially in the outpatient setting, as we continue to deliver the same therapies and treatment pathways that have relied on hospitalization for the past 30 years. This underscores the need for novel, practical, and accessible outpatient treatment strategies for ADHF.

## Outpatient decongestion barriers and limitations

Loop diuretics remain the mainstay of decongestion therapy. Still, their effectiveness can be reduced by inadequate dosing, reduced absorption of oral formulations, resistance to their pharmacologic actions, and compensatory changes in renal sodium reabsorption that often necessitate IV administration during ADHF. When loop diuretics are insufficient, adjuncts like thiazides, carbonic anhydrase inhibitors, or inotropes are used to boost diuresis, though, these combinations can increase electrolyte disturbances and renal dysfunction without consistently improving clinical outcomes. Recent outpatient studies have explored these strategies with mixed results (Table 1). In SALT-HF (2024), the addition of hypertonic saline to IV furosemide in 167 ambulatory worsening HF patients failed to improve short-term diuresis, congestion scores, or 30-day clinical outcomes compared to IV furosemide alone [3]. Conversely, the DEA-HF (2024) trial randomized 42 patients with worsening HF to combination regimens and demonstrated that IV furosemide plus oral metolazone produced significantly greater natriuresis and urinary output at six hours compared to IV furosemide alone or when combined with IV acetazolamide (Table 1) [4]. These data suggest that while some diuretic adjuncts may enhance natriuresis, results remain heterogeneous, highlighting the ongoing difficulty of achieving reliable, sustained decongestion in the outpatient setting. When these medical therapies are exhausted or unavailable, further treatment is limited to mechanical fluid removal with dialysis or other experimental invasive therapies that require hospitalization.

Beyond the pharmacologic challenges for successful decongestion with these agents, many logistical barriers prevent high throughput and effective outpatient management of ADHF. IV loop diuretics administered in the clinic are usually limited to a single dose or infusion, as arranging serial administrations is constrained by staffing, patient mobility, and insurance coverage. Sequential dosing of diuretics over multi-day periods can achieve potent fluid removal but requires close monitoring and patient compliance, raising safety concerns with heightened risk of complications. Consequently, hospitalization remains the default and most practical setting for decongestion, even among patients who may otherwise be safely managed in an outpatient clinic or at home.

**Table 1:** Key studies on decongestion and outpatient ADHF therapies

Key studies on decongestion and outpatient ADHF therapies					
Study	Population (n)	Patient setting	Intervention	Outcome	Key finding
AT HOME-HF (2024)	WHF patients (34)	Outpatient	Subcutaneous furosemide vs. IV loop diuretics	Win ratio of a 30-day hierarchical composite of cardiovascular death, HF events, and change in N-terminal pro–B-type natriuretic peptide	SC furosemide enhanced weight loss in congested HF patients. While the primary endpoint was not significant, improvements in dyspnea, function, and KCCQ-12 trends support further investigation as a hospitalization alternative
RED DESERT, SAHARA (2024)	ADHF patients with cardiorenal syndrome (8, 10)	Inpatient and outpatient	Direct sodium removal therapy (single arm)	Diuretic response, renal function, diuretic dose reduction	Direct sodium removal therapy improved diuretic responsiveness and cardiorenal parameters, allowing withdrawal of high-dose loop diuretics
Intranasal Bumetanide Study (2024)	Healthy subjects (68)	N/A	Intranasal bumetanide (bumetanide Nasal Spray; BNS) vs IV bumetanide vs oral bumetanide	Bioavailability, diuresis, natriuresis, safety	BNS had equivalent bioavailability to oral bumetanide, with faster absorption (Tmax 1 hr vs. 1.5 hrs PO). Diuresis was comparable across groups, with earlier natriuresis in BNS vs. IV. Fewer adverse events with BNS vs. PO. BNS may be a viable alternative for patients with poor GI absorption but further research in ADHF patients is needed.
SALT-HF (2024)	WHF patients (167)	Outpatient	IV furosemide plus hypertonic saline vs. IV furosemide	3-hr diuresis, congestion and renal function, 30-day clinical events	IV Furosemide plus hypertonic saline did not improve 3-hr diuresis or congestion parameters in patients with ambulatory WHF
DEA-HF (2024)	WHF patients (42)	Outpatient	IV furosemide vs. IV furosemide plus oral metolazone vs. IV furosemide plus IV acetazolamide	Sodium excretion and total urinary output at 6 hours	IV furosemide plus metolazone resulted in a significantly higher natriuresis compared with IV furosemide alone or furosemide plus acetazolamide
Ongoing Studies					
TROUPER (Tolvaptan For Worsening Outpatient Heart Failure: Role of Copeptin In Identifying Responders; NCT02476409	WHF patients (target: 40)	Outpatient	Augmentation of current diuretic dose with tolvaptan vs. placebo	Change in bodyweight at 48-hrs	Enrolling
A Phase 2, Double-Blind, Placebo-Controlled, Dose Range Finding Study to Assess Safety, Tolerability, Efficacy and Pharmacokinetics of the Relaxin Agonist R2R01 Combined With Standard of Care Versus Standard of Care Alone in Outpatients With Worsening Heart Failure (WHF); NCT06264310	WHF patients (target: 32)	Outpatient	Increasing doses of relaxin agonist R2R01 vs. placebo	NT-proBNP, renal function, and 30-day clinical events	Enrolling

To overcome these challenges, several outpatient trials are testing novel pharmacologic approaches. The TROUPER trial (NCT02476409) is evaluating whether adding tolvaptan to standard diuretic therapy, guided by copeptin levels, improves 48-hour weight loss in worsening HF outpatients [5]. Likewise, a phase 2 dose-finding study of the relaxin agonist R2R01 (NCT06264310**)** is enrolling outpatients with worsening HF to assess whether this vasodilatory peptide can enhance natriuresis and preserve renal function compared with standard care Table 1) [6]. Together, these efforts highlight a growing shift toward safe, scalable, physiology-guided outpatient therapies aimed at reducing HF admissions.

## Emerging outpatient therapy options

While many barriers exist to the successful management of ADHF in an outpatient setting, recent innovations have begun to close the gap between inpatient and ambulatory ADHF management. In the AT HOME-HF pilot trial (2024), the subcutaneous furosemide pump (Furoscix™) was compared with conventional IV loop diuretics for decongestion in worsening HF outpatients (*n* = 34).^7^ Although the primary composite endpoint – a 30-day hierarchical win ratio of cardiovascular (CV) death, HF events and NT-proBNP change – was not statistically significant, subcutaneous administration achieved enhanced weight loss and improvements in dyspnea, New York Heart Association (NYHA) functional status, and Kansas City Cardiomyopathy Questionnaire (KCCQ) score (Table 1). In October 2022, Furoscix was FDA-approved for the treatment of congestion due to fluid overload in adult NYHA Class II-III chronic HF patients, as a subcutaneous dose equivalent to an IV dose of 80mg [8]. In March 2025, the FDA expanded the indication to include treating edema in CKD patients based on pharmacokinetic and pharmacodynamic data.

Similarly, Enbumyst™ (intranasal bumetanide), was recently FDA-approved in 2025 as a loop diuretic for edema due to HF, liver or kidney disease [9]. In a phase 2 pharmacokinetic study of 68 healthy patients, it showed equivalent bioavailability to oral bumetanide and faster absorption (Tmax ~ 1 h vs. 1.5 h). Natriuresis onset was faster than IV administration, with fewer adverse events than oral formulation, suggesting Embumyst may be particularly valuable for patients with poor gastrointestinal absorption or limited IV access (Table 1). These novel therapies exemplify a patient-centered approach, reducing reliance on single-dose IV diuretics and frequent clinic visits by enabling at-home, multi-dose administration [7, 10]. 

## Device and implementation strategies

Despite advancements in loop diuretic formulations, resistance to diuretics remains a challenge. The Alfapump^®^ is a surgically implanted device that transfers sodium-free dialysate into the peritoneal cavity, creating a gradient for direct sodium removal [11]. In the RED DESERT and SAHARA feasibility studies (*n* = 8 and 10, respectively), this approach improved diuretic responsiveness and cardiorenal parameters, allowing withdrawal of high-dose loop diuretics in patients with diuretic resistance (Table 1). While promising for improving diuretic responsiveness, it remains an investigational therapy unsuitable for rapid outpatient ADHF relief.

Pulmonary artery pressure monitoring devices (CardioMEMS™ and Cordella™) are complementary devices, which lack intrinsic therapeutic potential, but enable clinicians to detect rising filling pressures and make timely diuretic adjustments potentially before diuretic resistance develops. Pooled data from large randomized controlled trials (CHAMPION, GUIDE-HF, and LAPTOP-HF) have demonstrated improvements in mortality and HF hospitalizations in patients with HFrEF. Integrating remote hemodynamic monitoring with novel pharmacologic options represents a synergistic opportunity to build a precision, home-based care model for decongestion [12]. Further research will be needed to determine how to effectively pair monitoring strategies with novel therapeutics in an outpatient setting (i.e. how to set cutoffs for escalation of therapy), similar to ongoing clinical trials evaluating PA pressure thresholds and need for device therapies (TEAM-HF) [13]. 

**Fig. 1:**
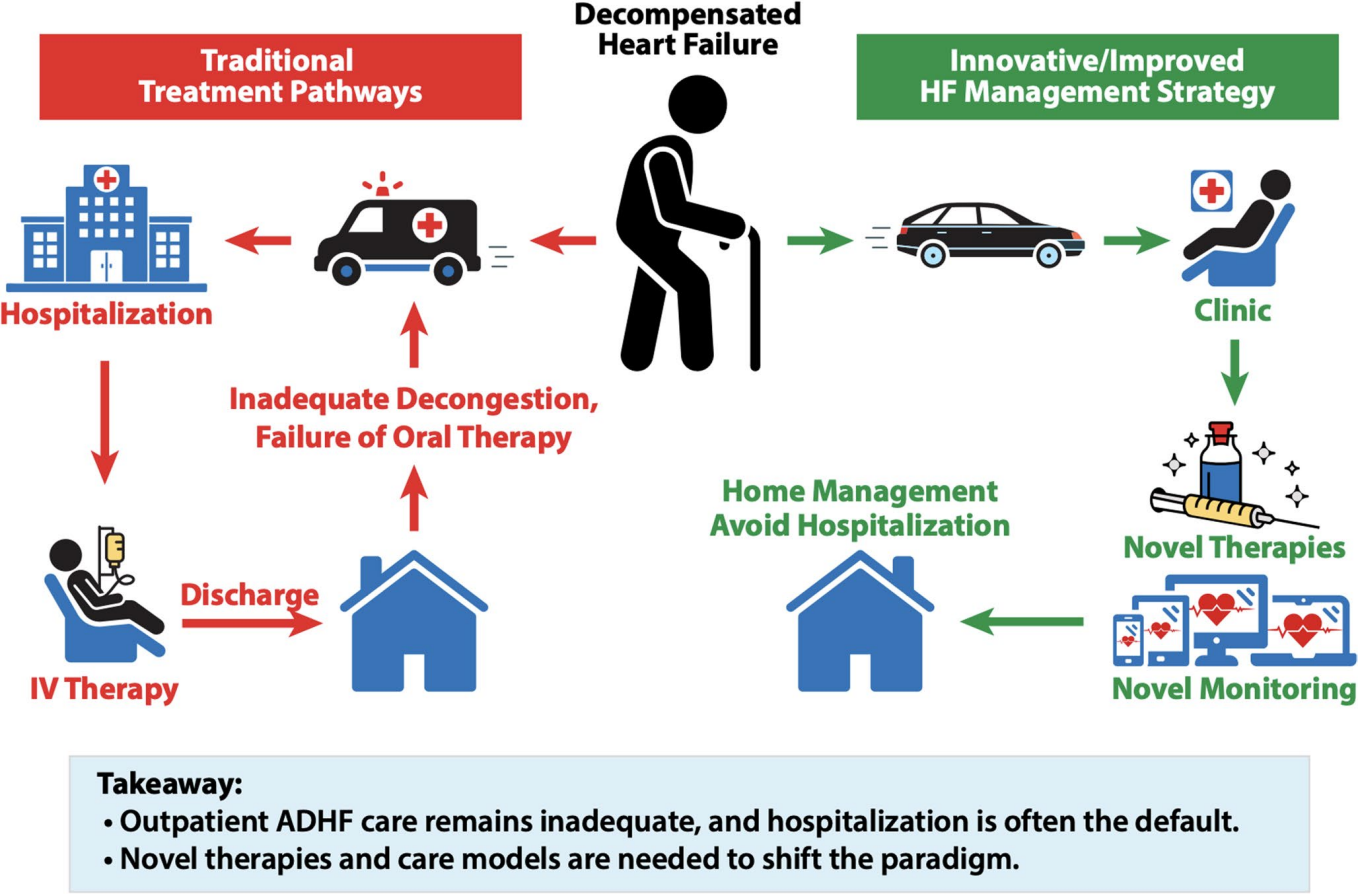
Traditional hospital based pathways for worsening heart failure management versus potential future at-home treatment pathways

The growing prevalence of outpatient ADHF underscores the urgent need for scalable management strategies and new treatment pathways (Fig. 1). Despite recent therapeutic advances, continued innovation is essential to break the cycle of decompensation and readmission and to reduce the burden on patients and the healthcare system. The next steps will require rigorous implementation studies, cost-effectiveness analyses, and health-system redesign to define how these therapies can be delivered safely, efficiently, and equitably. Integrating novel pharmacologic agents, device-based tools, and remote monitoring into coordinated care models, such as nurse- and pharmacist-led diuretic clinics, could be transformative in the struggle to keep HF patients out of the hospital while improving survival and quality of life. As these care models evolve, the answer to the question ‘Do I really need to be admitted?’ will increasingly, and appropriately, become ‘Not necessarily.’

## Data Availability

No datasets were generated or analysed during the current study.
